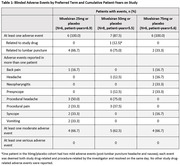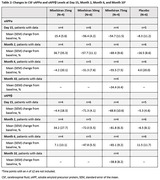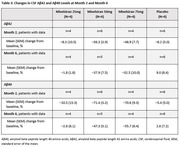# Single ascending dose results from an ongoing Phase 1 study of mivelsiran (ALN‐APP), the first investigational RNA interference therapeutic targeting amyloid precursor protein for Alzheimer’s disease

**DOI:** 10.1002/alz.084521

**Published:** 2025-01-09

**Authors:** Sharon Cohen, Simon Ducharme, Jared R. Brosch, Everard G.B. Vijverberg, Alexandre Sostelly, Sasikiran Goteti, Lynn Farrugia, Andreja Avbersek, Cristin Kaspar, Catherine J. Mummery

**Affiliations:** ^1^ Toronto Memory Program, Toronto, ON Canada; ^2^ Douglas Mental Health University Institute, Department of Psychiatry, McGill University, Montreal, QC Canada; ^3^ Indiana University School of Medicine, Indianapolis, IN USA; ^4^ Alzheimer Center Amsterdam, Amsterdam UMC, Vrije Universiteit Amsterdam, Amsterdam Netherlands; ^5^ Alnylam Pharmaceuticals, Cambridge, MA USA; ^6^ Regeneron Pharmaceuticals Inc., Tarrytown, NY USA; ^7^ University College London, London United Kingdom

## Abstract

**Background:**

Mivelsiran (ALN‐APP) is an investigational, intrathecally administered RNA interference therapeutic designed to lower levels of amyloid‐β (Aβ) peptide, a key driver of Alzheimer’s disease (AD) and cerebral amyloid angiopathy (CAA) pathogenesis, by reducing upstream production of amyloid precursor protein (APP). We report additional safety, pharmacodynamic, and biomarker data from the double‐blind, placebo‐controlled, single ascending dose part of the ongoing mivelsiran Phase 1 study (NCT05231785).

**Method:**

Patients with early‐onset AD (symptom onset <65 years of age, Clinical Dementia Rating global score 0.5 or 1.0, and Mini‐Mental State Examination score >20) were randomized to single intrathecal doses of mivelsiran (25mg, 50mg, or 75mg) or placebo and evaluated for 6 months (plus up to 6‐months follow‐up if needed to achieve washout). Primary endpoint was frequency of adverse events (AEs). Pharmacological effects of mivelsiran (secondary endpoints) and exploratory biomarkers of disease progression were also evaluated.

**Result:**

As of November 16, 2023, 20 patients (mean [range] age, 61.3 [53–73] years; 60.0% male; 80.0% white) were randomized to mivelsiran or placebo in 25mg (N = 6, 2:1 randomization), 50mg (N = 8, 3:1), and 75mg (N = 6, 2:1) cohorts. In these pooled cohorts, AEs were reported in 19 patients (95.0%), all of mild or moderate severity (Table 1). One patient experienced two mild AEs that were considered both study drug and procedure related. No serious AEs or deaths occurred. Reductions from baseline in cerebrospinal fluid (CSF) soluble APPα and APPβ levels were rapid and sustained through ongoing data capture (i.e., Month 6 with mivelsiran 50mg and Month 10 with mivelsiran 75mg; Table 2), and were accompanied by sustained reductions from baseline in CSF Aβ42 and Aβ40 levels available through Month 6 (Table 3). Safety and pharmacodynamic data of up to 6 months from additional mivelsiran dose cohorts will be presented at the meeting.

**Conclusion:**

In this ongoing Phase 1 single ascending dose study, mivelsiran 50mg and 75mg were well tolerated and produced robust, durable reductions in CSF levels of soluble APP and downstream Aβ42 and Aβ40, key proteins implicated in progression of AD and CAA. These interim results support further evaluation of mivelsiran in patients with AD or CAA.